# The Influence of Family Socioeconomic Status on Primary School Students’ Emotional Intelligence: The Mediating Effect of Parenting Styles and Regional Differences

**DOI:** 10.3389/fpsyg.2022.753774

**Published:** 2022-02-21

**Authors:** Zixiao Liu, Guohong Wu

**Affiliations:** ^1^Department of Psychology, School of Social Development and Public Policy, Fudan University, Shanghai, China; ^2^School of Humanities, Tongji University, Shanghai, China

**Keywords:** primary school students, family socioeconomic status, parenting style, emotional intelligence, mediating effect

## Abstract

Select 180 primary school students from a city primary school in Shanghai, a developed area in eastern China, and 146 primary school students from a rural primary school in Jingzhou, a centrally underdeveloped area, as subjects. The method of scale is used to explore the influence of family socioeconomic status on the emotional intelligence of primary school students, and the mediating role of parenting styles in this influence and the difference in this effect in the two regions. The results show that: (1) The socioeconomic status and emotional intelligence of primary school students in Jingzhou are significantly lower than those of primary school students in Shanghai. In terms of parenting style, the emotional warmth and understanding of the fathers and mothers of Jingzhou’s primary school students are both significantly lower than those of Shanghai’s primary school students; (2) the socioeconomic status and emotional intelligence of the primary school students in Jingzhou are significantly and positively correlated with the parents’ emotional warmth and understanding parenting styles, while the socioeconomic status and emotional intelligence of the primary school students in Shanghai are only significantly positively correlated with the father’s emotional warmth and understanding parenting style; and (3) parenting style has a mediating effect between family socioeconomic status and emotional intelligence, but this effect has regional differences. The specific performance is as follows: The parents’ emotional warmth and understanding parenting styles of the primary school students in Jingzhou play a partial mediating effect between the family socioeconomic status and emotional intelligence, while the Shanghai primary school students’ fathers’ emotional warmth and understanding parenting style plays a complete mediating effect in family socioeconomic status and emotional intelligence.

## Introduction

Since the reform and opening up, Chinese social economy has developed rapidly, and people’s living standards have continued to improve, but at the same time, there have also been problems, such as imbalanced urban and rural social and economic development. This imbalance leads to differentiation and stratification of family socioeconomic status (Liu and Tian, 2018). Psychologists, especially the developmental psychologists, generally research the children’s development from the family perspective, so they became interested in the indicator of family socioeconomic status, and began to explore its possible impact on the development of children.

Existing studies have found that family socioeconomic status has a significant impact on children’s and adolescents’ learning investment, academic performance, intelligence, internal motivation, and creativity (Hanushek, 1986; Woessmann, 2003; Shi and Shen, 2007), while low family socioeconomic status will have a negative impact on the development of children’s social emotions and social interactions, and these children are more likely to suffer from social anxiety disorder (Zhu and Zhang, 2013). In addition to studying the direct impact of family socioeconomic status on children’s development, some scholars have also studied the indirect impact of family socioeconomic status on children’s development. For example, Su et al. (2015) found that the family socioeconomic status of left-behind children is significantly positively correlated with their social adaptation, and the perception of group discrimination plays a significant mediating role in this influence. In the choice of mediating variables, parenting styles are selected more frequently. This choice is not difficult to understand in the field of developmental psychology: on the one hand, there have been many Studies on the relationship between family socioeconomic status and parenting styles. It is generally found that family socioeconomic status is significantly positively correlated with parents’ positive parenting styles, while negatively correlated with negative parenting styles (Zhang, 2016; Luo et al., 2021); on the other hand, among the many factors that affect the development of children, the important role of parenting on children’s development has been supported by many theories and confirmed by research, such as the “attachment” theory and the strange situation experiment (Ainsworth and Wittig, 1969; Bowlby, 1969).

Therefore, family socioeconomic status can affect children’s development through parenting style, which are supported by both logical reasoning and empirical materials, such as migrant children’s cognitive ability (Zhang et al., 2017a), middle school students’ academic performance (Li, 2013), the awareness of children’s rights (Gong et al., 2012), belief in a just world (Zhang et al., 2017a), and Children’s learning quality (Ye et al., 2021), and other fields have been studied by researchers.

While for emotional intelligence, an intelligence factor that is explicitly proposed in the 1990s (Mayer et al., 1990). Since the emotional intelligence theory is put forward, after nearly 30 years of development, a large number of definitions and theoretical models of emotional intelligence have emerged. At present, different scholars have different classifications of emotional intelligence. Among them, Chen et al. (2012) divides emotional intelligence into four main types. The first is ability model represented by Mayer et al. (2000), who think emotional intelligence is an intelligence similar to analytical intelligence, and they divide emotional intelligence into four dimensions: emotion perception and expression, integrating emotions into thinking, understanding and analyzing emotions, and reflective emotion management. The second is mixed model represented by Bar-on (2006), who considers emotional intelligence is a series of skills and traits that help people adapt to social and emotional needs in life, and he divides emotional intelligence into five dimensions: individual dimension, interpersonal dimension, stress management dimension, adaptability dimension, and general mood dimension. The third is competence model represented by Goleman (1995), who believes emotional intelligence is a series of emotional and social competency traits associated with outstanding performance in the workplace, he also divides emotional intelligence into five dimensions: knowing one’s own emotions, manage one’s own emotions, self-motivation, knowing the emotions of others, and handling interpersonal relationships. And the last is trait model represented by Petrides and Furnham (2001), who insist emotional intelligence is a series of personality traits, they divide emotional intelligence into four dimensions: emotional, social, self-control, and wellbeing. Based on the definition and classification of emotional intelligence by these scholars, the main controversy about emotional intelligence is whether it is an ability, a trait, or a combination of the two. According to this classification, Salovey and Mayer is ability model, Furnham and Petrides is trait model, and Bar-on and Gorman are mixed models.

Putting aside the internal disputes among scholars on the definition of the concept of emotional intelligence for the time being, no matter whose theory of emotional intelligence is adopted, many studies have shown that parenting styles are significantly related to emotional intelligence (Joseph et al., 2002; Wang and He, 2002; Al-Elaimat et al., 2020; Nguyen et al., 2020; Lu, 2021), but further research is still lacking. While emotional intelligence is significantly positively correlated with prosocial behavior, life adaptation, mental health, work performance, and other factors and is significantly negatively correlated with problem behaviors, such as substance abuse, skipping classes, and dropping out of school, has been supported by a large number of studies (Mayer et al., 2004; Yang, 2008; Yang, 2011; Du et al., 2019). Therefore, further exploration of the family influence mechanism of emotional intelligence is of great significance to the children and adolescents’ mental health growth and adapt to society. In addition, Hansen (2003) found that the degree of social stratification will determine the degree of socioeconomic status (SES) effect. According to this, the influence mechanism of Chinese urban and rural families may be different by the degree of family social stratification. In summary, this study attempts to further explore the family influence mechanism of emotional intelligence. It is assumed that the family socioeconomic status of primary school students will affect their emotional intelligence through the mediating effect of parenting styles, but this mechanism has regional differences. Specifically, the hypotheses of this study are as follows:

1. There are significant regional differences in family socioeconomic status, parenting style, and emotional intelligence among primary school students.
2. The family socioeconomic status, parenting styles, and emotional intelligence of primary school students are significantly correlated with each other.
3. The family socioeconomic status of primary school students affects their emotional intelligence, and parenting style mediates the effect between family socioeconomic status and emotional intelligence, but there are regional differences in this effect.

## Materials and Methods

### Participants

Select one urban primary school in Shanghai (hereinafter referred to as Shanghai) and one rural primary school in Jingzhou Miao and Dong Autonomous County of Hunan Province (hereinafter referred to as Jingzhou). Considering that there are certain requirements for questionnaire cognition and reading ability, based on the research experience of previous researchers (Liu and Zhou, 2009), students in the first and second grades of primary school are excluded. Four hundred questionnaires are distributed, and 326 valid questionnaires are obtained after excluding the questionnaires with a missing rate of more than 50% and the obvious regular response. Among them, there are 180 primary school students in Jingzhou, with an average age of 9.71 years (SD = 0.71), 89 boys and 91 girls; 146 primary school students in Shanghai, with an average age of 10.22 years (SD = 0.87), 72 boys and 74 girls.

### Research Tools

#### Emotional Intelligence Scale

Due to the different theoretical orientations, the scale design is also different. The author believes that it is better to choose a mixed model of comprehensive orientation before there is sufficient evidence to show that emotional intelligence is just a single orientation of ability or traits. As for the selection of Goleman’s and Bar-on’s theories of emotional intelligence, As for the selection of Goleman’s and Bar-on’s theories of emotional intelligence, Goleman’s theory of emotional intelligence is chosen as the guiding theory for the measurement of emotional intelligence in this study because on the one hand, its 5D, compared to Bar-on’s 5D, are more focused on the emotional dimension, on the other hand, his theory has been used as a guiding theory by Chinese scholars to develop a scale suitable for measuring emotional intelligence in primary school students with good reliability and validity (Qiu, 2008). Therefore, select Qiu’s Emotional Intelligence Scale for primary school students based on Goleman’s theory of emotional intelligence, translated by Yang into a simplified Chinese version (Goleman, 1995; Qiu, 2008; Yang, 2011), its internal consistency coefficient is 0.92. This scale has been tested in Chinese primary school groups, which is superior to other scales in terms of cultural adaptability and applicability to the primary school groups. The scale includes five dimensions: knowing one’s own emotions, manage one’s own emotions, self-motivation, knowing the emotions of others, and handling interpersonal relationships. Using the Likert five-point scoring method, there are 23 questions in total. The higher the score, the higher the level of emotional intelligence of the subject. In this study, the internal consistency coefficient of the scale is 0.93.

#### Parenting Style Scale

Since, Yue (1993) did a lot of research in China based on the EMBU scale and adapted the EMBU scale to the actual situation in China, many Chinese scholars have also adopted their modified scales, and all of these studies have confirmed that their scales have good reliability and validity. Furthermore, the differences in parenting styles between father and mother have been proven in many studies (Yang, 2015; Du, 2021), the scale requires separate responses on the parenting style of the father and mother, which could help us to discuss separately the role played by father’s and mother’s parenting styles in the relationship between family socioeconomic status and emotional intelligence in primary school students. Therefore, select the revised EMBU scale based on the study of the Chinese population by Yue (1993). The parenting style of the father on the scale is divided into 6 dimensions: emotional warmth and understanding, punishment and harshness, excessive interference, overprotection, favoring subject, rejection and denial; the parenting style of mother is divided into 5 dimensions: emotional warmth and understanding, punishment and harshness, excessive interference and overprotection, favoring subject, rejection and deny. Since children in this age group are born during the one-child policy period and the two-child policy has just been implemented, the dimension that preference for subjects is deleted. Using the Likert four-point scoring method, a total of 62 questions. The higher the score on a dimension, the more often the subject’s parents used this parenting style. In this study, the internal consistency coefficient of the scale is 0.93.

#### Family Socioeconomic Status Scale

The selection of the family socioeconomic status scale is more complicated. Theoretically, it is more comprehensive to measure the three indicators of parents’ education, income, and occupation. However, due to the evaluation of these three concepts by the primary school students, there may be greater deviations. To solve this problem, the Program for International Student Assessment (PISA) and the World Health Organization both use household goods as a measure of family income (Currie et al., 1997). The PISA project has been implemented in China for many years. Some scholars have also used it for research in China based on the compilation of a household goods scale. The research results have good reliability and validity (Ren, 2010; Ren and Xin, 2013). The research of Currie et al. (1997) also showed that the response rate of the student group to parents’ education and parents’ occupation is significantly lower than that of household goods. Therefore, in summary, this study decided to take the household goods scale designed by Ren and Xin (2013) according to the PISA project as the blueprint. The design includes three aspects of the family’s material living conditions, regular learning conditions, and spiritual and cultural life objects, as a primary school student’s family socioeconomic status research tools. Using 0,1 scoring method, 12 questions included. The higher the score, the higher the family socioeconomic status of the subject.

### Research Procedures

An ethical application was made for this study with approval number FDU-SSDPP-IRB-2021\1-028. The test is conducted in an anonymous manner. The author and other psychology students serve as the main test. Students will be informed of the following instructional phrases uniformly before the test: Hello, thank you very much for taking part in this survey, this questionnaire is designed to complete a scientific study and to understand some of your growing experience, all questions are not “right” or “wrong,” please fill in or select the appropriate answer according to your own real situation and feelings. Please feel free to answer, we will protect your privacy, all answers will not bring any adverse effects to your study and life. There is no monetary reward or credits for participation. The questionnaire is collected on the spot.

### Data Analysis Methods

The data are analyzed using SPSS18.0, where *t*-test is used for difference comparison, Pearson’s *r* is used for correlation analysis, and hierarchical regression analysis is used for mediating effect.

## Results

### Comparison of Differences in Family Socioeconomic Status, Parental Rearing Styles, and Emotional Intelligence of Primary School Students in Different Regions

#### Comparison of Differences in Family Socioeconomic Status and Emotional Intelligence of Primary School Students in Different Regions

It can be seen from Table 1 that by comparing the family socioeconomic status and emotional intelligence of primary school students in the two regions, the family socioeconomic status (*t* = −15.26), and emotional intelligence (*t* = −13.95) of Jingzhou primary school students are significantly lower than those of Shanghai primary school students (*p* < 0.001).

**Table 1:** Comparison of differences in family socioeconomic status and emotional intelligence of primary school students in different regions M (SD).

	Family Socioeconomic Status	Emotional Intelligence
Jingzhou (*n* = 180)	5.49 (2.19)	73.37 (11.88)
Shanghai (*n* = 146)	9.20 (2.18)	93.73 (14.02)
*t*	−15.26^***^	−13.95^***^

*** *p* < 0.001.

#### Comparison of Differences in Parenting Styles of Fathers of Primary School Students in Different Regions

According to Table 2, in comparison of regional differences in fathers’ parenting styles, Jingzhou primary school students’ fathers’ emotional warmth and understanding (*t* = −8.52) is significantly lower than those of Shanghai primary school students (*p* < 0.001); in addition, Jingzhou primary school students’ fathers’ excessive interference (*t* = −2.23) is also significantly lower than Shanghai primary school students (*p* < 0.05). In the other three dimensions of father’s parenting style, punishment and harshness (*t* = 2.07), overprotection (*t* = 4.59), rejection and denial (*t* = 2.17), and Jingzhou primary school students are significantly higher than the Shanghai primary school students (*p* < 0.05, *p* < 0.001, *p* < 0.05).

**Table 2:** Comparison of differences in parenting styles of primary school students’ fathers in different regions M (SD).

	Parenting Styles of Fathers				
	Emotional Warmth and Understanding	Punishment and Harshness	Excessive Interference	Overprotection	Rejection and Denial
Jingzhou (*n* = 180)	50.35 (7.48)	22.06 (7.01)	21.26 (4.05)	12.26 (2.84)	10.81 (3.49)
Shanghai (*n* = 146)	59.20 (10.60)	20.35 (7.86)	22.45 (5.30)	10.73 (3.13)	9.93 (3.88)
*t*	−8.52^***^	2.07^*^	−2.23^*^	4.59^***^	2.17^*^

* *p* < 0.05;

*** *p* < 0.001.

#### Comparison of Differences in Parenting Styles of Mothers of Primary School Students in Different Regions

According to Table 3, in the regional comparison of mothers’ parenting styles, Jingzhou primary school students’ mothers’ emotional warmth and understanding (*t* = −4.73) are also significantly lower than those of Shanghai primary school students (*p* < 0.001), which is the same with the regional differences in father’s parenting styles. The difference is that in the other three dimensions of the mother’s parenting style: punishment and harshness, excessive interference and overprotection, rejection and denial, there is no significant difference between the two regions (*p* > 0.05).

**Table 3:** Comparison of differences in the parenting styles of mothers of primary school students in different regions M (SD).

	Parenting Styles of Mothers			
	Emotional Warmth and Understanding	Punishment and Harshness	Excessive Interference and Overprotection	Rejection and Denial
Jingzhou (*n* = 180)	53.44 (7.85)	15.72 (4.80)	37.65 (5.86)	14.60 (3.92)
Shanghai (*n* = 146)	58.97 (12.22)	14.68 (6.78)	36.53 (7.37)	13.86 (5.73)
*t*	−4.73^***^	1.58	1.50	1.33

*** *p* < 0.001.

### Correlation Research on Primary School Students’ Family Socioeconomic Status, Parenting Styles, and Emotional Intelligence

#### Correlation Research on Primary School Students’ Family Socioeconomic Status, Father’s Parenting Style, and Emotional Intelligence

According to Table 4, it can be seen that the family socioeconomic status of primary school students in the two regions is significantly positively correlated with emotional intelligence (*r* = 0.23, *r* = 0.24, *p* < 0.01). In the relationship with the parenting style of the father, there are both similarity and difference in the two regions: The similarity is that the emotional warmth and understanding of the fathers of the primary school students in the two areas are both related to the family socioeconomic status (*r* = 0.18, *r* = 0.28, *p* < 0.05, *p* < 0.01) and emotional intelligence (*r* = 0.34, *r* = 0.53, *p* < 0.01, *p* < 0.001); the difference is that the other parenting styles of Jingzhou primary school fathers are not significantly related to family socioeconomic status and emotional intelligence, while the punishment and harshness (*r* = −0.26), rejection and denial (*r* = −0.27) by the father of Shanghai primary school students are significantly negatively correlated with their emotional intelligence (*p* < 0.01).

**Table 4:** The correlation between primary school students’ family socioeconomic status, father’s parenting style, and emotional intelligence.

		Family SES	Parenting Styles of Fathers				
			Emotional Warmth and Understanding	Punishment and Harshness	Excessive Interference	Overprotection	Rejection and Denial
Jingzhou (*n* = 180)	Family SES Emotional Intelligence	1 0.23^**^	0.18^*^ 0.34^**^	−0.04 −0.02	0.04 −0.04	0.08 0.12	−0.03 −0.01
Shanghai (*n* = 146)	Family SES Emotional Intelligence	1 0.24^**^	0.28^**^ 0.53^***^	−0.15 −0.26^**^	0.10 −0.04	0.04 −0.08	−0.08 −0.27^**^
Total (*n* = 326)	Family SES Emotional Intelligence	1 0.18^**^	0.23^**^ 0.36^**^	−0.08 −0.18^**^	0.08 0.08	0.06 −0.12^*^	−0.05 −0.18^**^

* *p* < 0.05;

** *p* < 0.01;

*** *p* < 0.001.

#### Correlation Research on Primary School Students’ Family Socioeconomic Status, Mother’s Parenting Style, and Emotional Intelligence

According to Table 5, we can find the relationship between the parenting style of primary school students’ mothers and their family socioeconomic status and emotional intelligence in the two regions. Jingzhou primary school students’ mothers’ emotional warmth and understanding are significantly positively related to their family socioeconomic status (*r* = 0.19, *p* < 0.05) and emotional intelligence (*r* = 0.32, *p* < 0.01). while the parenting styles of mothers in Shanghai primary school students are not significantly related to their family socioeconomic status, but their emotional intelligence and mother’s emotional warmth and understanding (*r* = 0.40, *p* < 0.01) and mother’s excessive interference and overprotection significantly positively correlated (*r* = 0.21, *p* < 0.05) and significantly negatively correlated (*p* < 0.05) with the mother’s punishment and harshness (*r* = −0.17).

**Table 5:** The correlation between primary school students’ family socioeconomic status, mother’s parenting style, and emotional intelligence.

		Family SES	Parenting Styles of Mothers			
			Emotional Warmth and Understanding	Punishment and Harshness	Excessive Interference and Overprotection	Rejection and Denial
Jingzhou (*n* = 180)	Family SES Emotional Intelligence	1 0.23^**^	0.19^*^ 0.32^**^	−0.05 −0.05	0.10 0.14	−0.05 −0.05
Shanghai (*n* = 146)	Family SES Emotional Intelligence	1 0.24^**^	0.15 0.40^**^	−0.02 −0.17^*^	0.14 0.21^*^	0.05 −0.15
Total (*n* = 326)	Family SES Emotional Intelligence	1 0.18^**^	0.15^**^ 0.41^**^	−0.03 −0.12^*^	0.13^*^ 0.12^*^	0.01 −0.12^*^

* *p* < 0.05;

** *p* < 0.01.

### The Mediating Effect of Primary School Students’ Parenting Style in the Influence of Family Socioeconomic Status on Emotional Intelligence

This study adopts the mediating effect test procedure proposed by Wen et al. (2004), and refers to the practice of Zhang et al. (2017b), and uses the method of hierarchical regression analysis to test the mediating effect.

#### The Mediating Effect of Shanghai Primary School Students’ Parenting Style in the Influence of Family Socioeconomic Status on Emotional Intelligence

**Figure 1:**
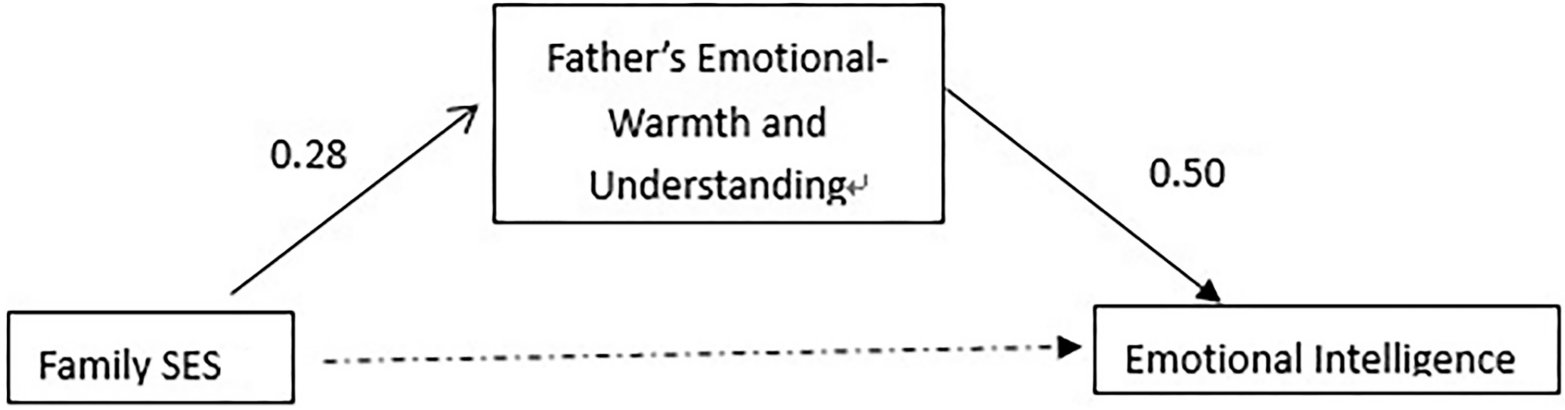
A model diagram of the mediating effect of father’s emotional warmth and understanding in Shanghai primary school students.

Using the method of hierarchical regression analysis, the family socioeconomic status of Shanghai primary school students and their father’s emotional warmth and understanding are processed centrally. Regression analysis of emotional intelligence to family socioeconomic status and father’s emotional warmth and understanding are carried out, respectively. The first lever puts the family socioeconomic status, and the second lever puts the father’s emotional warmth and understanding. The analysis results are as follows:

It can be seen from Table 6 that in the first-level regression, the family socioeconomic status of Shanghai primary school students has a significant predictive effect on emotional intelligence (*β* = 0.24, *p* < 0.01), and when the second-level regression adds the father’s emotional warmth and understanding at this time, family socioeconomic status is no longer significant in predicting emotional intelligence (*β* = 0.10, *p* > 0.05), and father’s emotional warmth and understanding has a significant predictive effect on emotional intelligence (*β* = 0.50, *p* < 0.001). Therefore, the emotional warmth and understanding of the fathers of Shanghai primary school students play a completely mediating effect in the influence of family socioeconomic status on emotional intelligence (Figure 1).

**Table 6:** Regression analysis of Shanghai primary school students’ emotional intelligence on family socioeconomic status, father’s emotional warmth and understanding.

Predictor Variable	Emotional Intelligence			
	*R* ^2^	Δ*R*^2^	*F*	*β*
**The First Level** Family SES	0.06	0.06	8.38^**^	0.24^**^
**The Second Level**	0.28	0.23	28.31^***^	
Family SES Father’s Emotional Warmth and Understanding				0.10 0.50^***^

** *p* < 0.01;

*** *p* < 0.001.

#### The Mediating Effect of Jingzhou Primary School Students’ Parenting Style in the Influence of Family Socioeconomic Status on Emotional Intelligence

**Figure 2:**
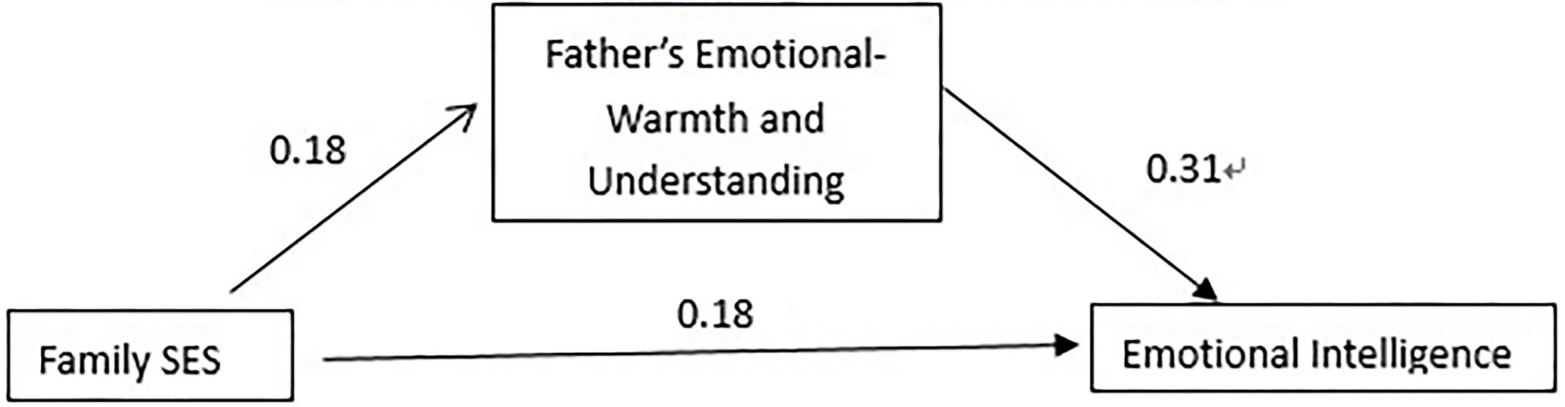
A model diagram of the mediating effect of father’s emotional warmth and understanding in Jingzhou primary school students.

Using the same method, the Jingzhou primary school students’ family socioeconomic status and father’s emotional warmth and understanding are processed centrally. The regression analysis of emotional intelligence to family socioeconomic status and father’s emotional warmth and understanding are carried out, respectively. The first level puts the family socioeconomic status, and the second level puts the father’s emotional warmth and understanding. The analysis results are as follows:

From the results in Table 7, in the first-level regression, the family socioeconomic status of Jingzhou primary school students has a significant predictive effect on emotional intelligence (*β* = 0.23, *p* < 0.01), when the second-level regression adds the father’s emotional warmth and understanding, father’s emotional warmth, and understanding also has a significant predictive effect on emotional intelligence (*β* = 0.31, *p* < 0.001), which is similar to that of Shanghai primary school students. The difference is that the family socioeconomic status at this time is still significant in predicting emotional intelligence (*β* = 0.18, *p* < 0.05). It shows that the father’s emotional warmth and understanding of Jingzhou primary school students plays a part of the mediating effect in the influence of family socioeconomic status on emotional intelligence. The total effect (β) of family socioeconomic status on emotional intelligence is 0.24, the direct effect is 0.18, and the proportion of indirect effects in the total effect is 0.18*0.31/0.24 = 0.23. Therefore, in this model, there is 23% of the influence of Jingzhou primary school students’ family socioeconomic status on emotional intelligence through the mediating effect of father’s emotional warmth and understanding (Figure 2).

**Table 7:** Regression analysis of Jingzhou primary school students’ emotional intelligence on family socioeconomic status, father’s emotional warmth, and understanding.

Predictor Variable	Emotional Intelligence			
	*R* ^2^	Δ*R*^2^	*F*	*β*
**The First Level** Family SES	0.05	0.05	10.25^**^	0.24^**^
**The Second Level**	0.15	0.09	15.30^***^	
Family SES Father’s Emotional Warmth and Understanding				0.18^*^ 0.31^***^

* *p* < 0.05;

** *p* < 0.01;

*** *p* < 0.001.

**Figure 3:**
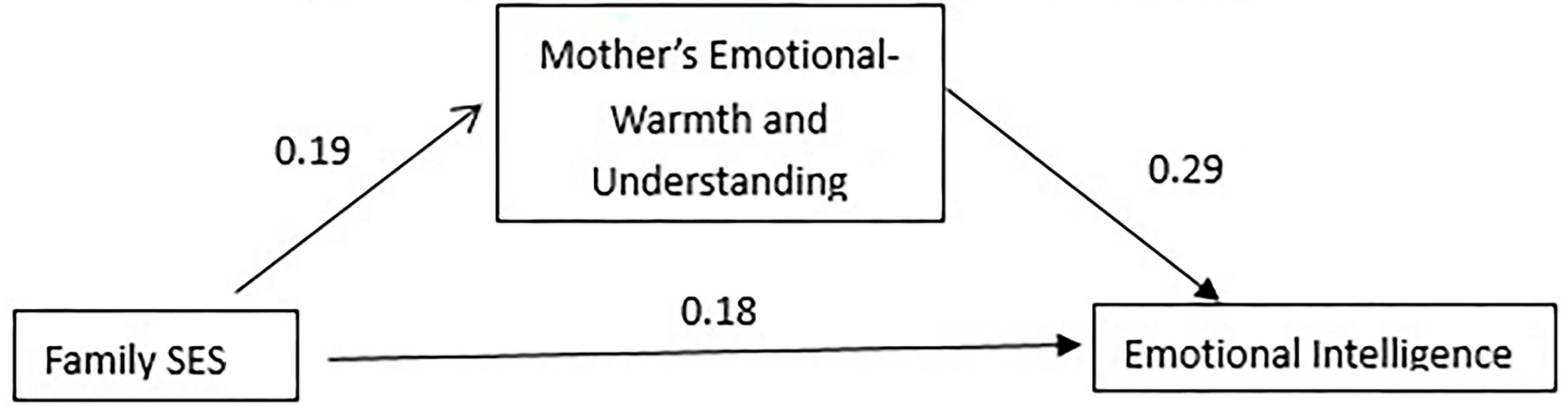
A model diagram of the mediating effect of mother’s emotional warmth and understanding in Jingzhou primary school students.

Using the same method, the Jingzhou primary school students’ family socioeconomic status and mother’s emotional warmth and understanding are processed centrally. The regression analysis of emotional intelligence to family socioeconomic status and mother’s emotional warmth and understanding are carried out, respectively. The first level puts the family socioeconomic status, and the second level puts the mother’s emotional warmth and understanding. The analysis results are as follows:

From the results in Table 8, in the first-level regression, the family socioeconomic status of Jingzhou primary school students has a significant predictive effect on emotional intelligence (*β* = 0.23, *p* < 0.01), when the second-level regression adds the mothers emotional warmth and understanding, mother’s emotional warmth, and understanding also has a significant predictive effect on emotional intelligence (*β* = 0.29, *p* < 0.001), while family socioeconomic status at this time still has a significant predictive effect on emotional intelligence (*β* = 0.18, *p* < 0.05). It shows that the mother’s emotional warmth and understanding of Jingzhou primary school students also plays a part of the mediating effect in the influence of family socioeconomic status on emotional intelligence. The total effect (*β*) of family socioeconomic status on emotional intelligence is 0.23, the direct effect is 0.18, and the proportion of indirect effects in the total effect is 0.18^*^0.29/0.23 = 0.23. Therefore, in this model, there is 23% of the influence of Jingzhou primary school students’ family socioeconomic status on emotional intelligence through the mediating effect of mother’s emotional warmth and understanding (Figure 3).

**Table 8:** Regression analysis of Jingzhou primary school students’ emotional intelligence on family socioeconomic status, mother’s emotional warmth, and understanding.

Predictor Variable	Emotional Intelligence			
	*R* ^2^	Δ*R*^2^	*F*	*β*
**The First Level** Family SES	0.05	0.05	10.25^**^	0.23^**^
**The Second Level**	0.13	0.08	13.63^***^	
Family SES Mother’s Emotional Warmth and Understanding				0.18^*^ 0.29^***^

* *p* < 0.05;

** *p* < 0.01;

*** *p* < 0.001.

#### The Mediating Effect of Total Primary School Students’ Parenting Style in the Influence of Family Socioeconomic Status On Emotional Intelligence

**Figure 4:**
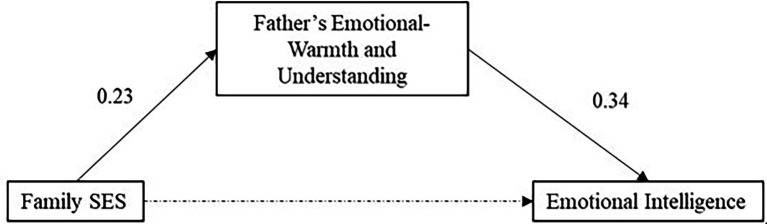
A model diagram of the mediating effect of father’s emotional warmth and understanding in total primary school students.

Using the same method, the total primary school students’ family socioeconomic status and father’s emotional warmth and understanding are processed centrally. The regression analysis of emotional intelligence to family socioeconomic status and father’s emotional warmth and understanding are carried out, respectively. The first level puts the family socioeconomic status, and the second level puts the father’s emotional warmth and understanding. The analysis results are as follows:

It can be seen from Table 9 that in the first-level regression, the family socioeconomic status of total primary school students has a significant predictive effect on emotional intelligence (*β* = 0.18, *p* < 0.01), and when the second-level regression adds the father’s emotional warmth and understanding at this time, family socioeconomic status is no longer significant in predicting emotional intelligence (*β* = 0.10, *p* > 0.05), and father’s emotional warmth and understanding has a significant predictive effect on emotional intelligence (*β* = 0.34, *p* < 0.001). Therefore, the emotional warmth and understanding of the fathers of total primary school students play a completely mediating effect in the influence of family socioeconomic status on emotional intelligence (Figure 4).

**Table 9:** Regression analysis of total primary school students’ emotional intelligence on family socioeconomic status, father’s emotional warmth, and understanding.

Predictor Variable	Emotional Intelligence			
	*R* ^2^	Δ*R*^2^	*F*	*β*
**The First Level** Family SES	0.03	0.03	10.98^**^	0.18^**^
**The Second Level**	0.14	0.11	26.24^***^	
Family SES Father’s Emotional Warmth and Understanding				0.10 0.34^***^

** *p* < 0.01;

*** *p* < 0.001.

**Figure 5:**
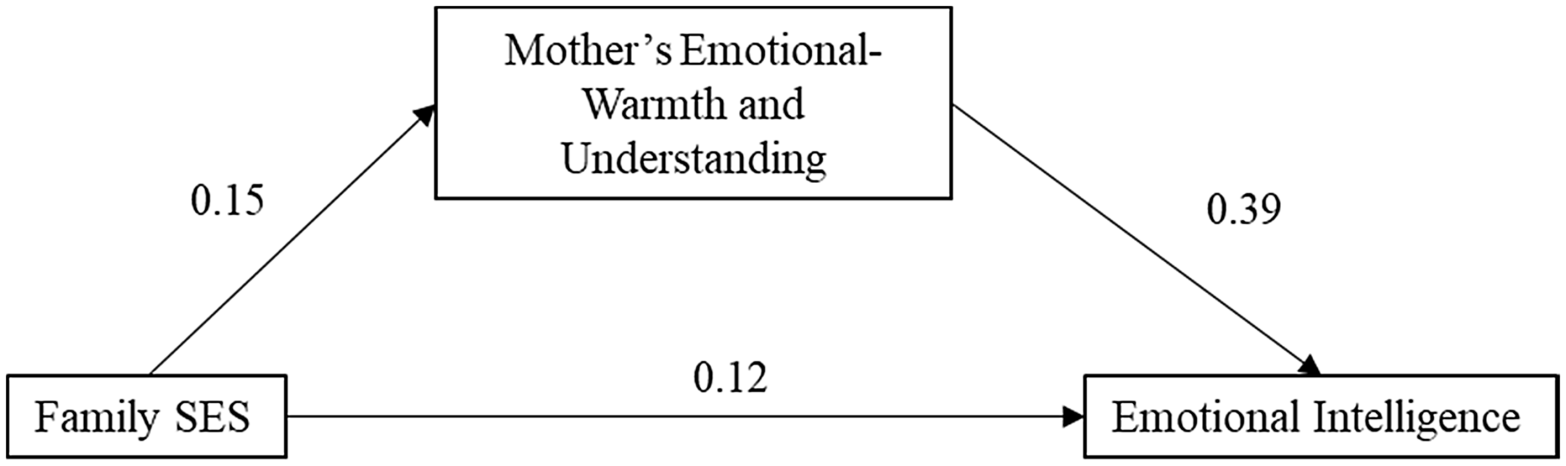
A model diagram of the mediating effect of mother’s emotional warmth and understanding in total primary school students.

Using the same method, the total primary school students’ family socioeconomic status and mother’s emotional warmth and understanding are processed centrally. The regression analysis of emotional intelligence to family socioeconomic status and mother’s emotional warmth and understanding are carried out, respectively. The first level puts the family socioeconomic status, and the second level puts the mother’s emotional warmth and understanding. The analysis results are as follows:

From the results in Table 10, in the first-level regression, the family socioeconomic status of total primary school students has a significant predictive effect on emotional intelligence (*β* = 0.18, *p* < 0.01), when the second-level regression adds the mothers emotional warmth and understanding, mother’s emotional warmth, and understanding also has a significant predictive effect on emotional intelligence (*β* = 0.39, *p* < 0.001), while family socioeconomic status at this time still has a significant predictive effect on emotional intelligence (*β* = 0.12, *p* < 0.05). It shows that the mother’s emotional warmth and understanding of total primary school students plays a part of the mediating effect in the influence of family socioeconomic status on emotional intelligence. The total effect (β) of family socioeconomic status on emotional intelligence is 0.18, the direct effect is 0.12, and the proportion of indirect effects in the total effect is 0.12*0.39/0.18 = 0.26. Therefore, in this model, there is 26% of the influence of total primary school students’ family socioeconomic status on emotional intelligence through the mediating effect of mother’s emotional warmth and understanding (Figure 5).

**Table 10:** Regression analysis of total primary school students’ emotional intelligence on family socioeconomic status, mother’s emotional warmth, and understanding.

Predictor Variable	Emotional Intelligence			
	*R* ^2^	Δ*R*^2^	*F*	*β*
**The First Level** Family SES	0.03	0.05	10.98^**^	0.18^**^
**The Second Level**	0.18	0.15	35.20^***^	
Family SES Mother’s Emotional Warmth and Understanding				0.12^*^ 0.39^***^

* *p* < 0.05;

** *p* < 0.01;

*** *p* < 0.001.

## Discussion

### Discussion on Differences in Family Socioeconomic Status, Parenting Styles and Emotional Intelligence of Primary School Students in Different Regions

#### Difference Comparison Meet the Hypothesis

From the research results, there are indeed differences in the family socioeconomic status, parental rearing styles, and emotional intelligence of primary school students in different regions. The family socioeconomic status of the students of Jingzhou rural primary schools is significantly lower than that of the students of Shanghai urban primary schools. This result is in line with the overall status quo of the imbalanced social and economic development in urban and rural areas after China’s reform and opening up, and the overall situation of rural areas lagging behind cities. The difference in family socioeconomic status will lead to differences in parenting styles. Parents with higher family economic status tend to use more active parenting styles, while parents with lower family economic status tend to use negative styles. In this study, Jingzhou primary school students with lower family socioeconomic status have significantly lower emotional warmth and understanding than Shanghai primary school students, and their fathers’ punishment and harshness, rejection and denial are significantly higher than those of Shanghai primary school students, which is also in line with this general view. Similarly, it is a consensus in developmental psychology that parenting styles affect children’s development. Studies have generally found that positive parenting styles are positively related to children’s emotional intelligence, while negative parenting styles are negatively related to children’s emotional intelligence (Joseph et al., 2002; Wang and He, 2002). The emotional intelligence of Jingzhou primary school students is significantly lower than that of Shanghai primary school students, which is also reasonable.

#### In Addition to Family Factors, School Factors Should Also Be Studied

In Goleman’s emotional intelligence questionnaire, a considerable amount of cognitive ability is also included. Yan (2017) studied the cognitive level of left-behind children in rural areas, children in rural intact families, migrant children who followed their parents to school in cities, and urban children. The results found that the cognitive level of urban children (including migrant children and urban children) are significantly higher than rural children (including left-behind and non-left-behind children); among urban children, the cognitive level of migrant children is still lower than that of urban children. It can be seen that in his research, family socioeconomic status does have an impact on cognitive level, but better education in urban schools also has a greater impact on children’s cognitive development. The questionnaire screening in this study also confirmed this difference. The third-grade primary school students in Jingzhou primary school had a very low rate of accurate response to the questionnaire, reflecting that they did not know many characters. However, the third-grade primary school students in Shanghai are able to respond smoothly, and the literacy level of the two is significantly different, the difference in literacy level is obvious due to the difference in school education. Therefore, in this study, the difference in emotional intelligence of primary school students of the same school age is so significant. In addition to the influence of family, the factors of school education should also be taken into consideration.

### Discussion on the Correlation Between Primary School Students’ Family Socioeconomic Status, Parenting Styles, and Emotional Intelligence

#### Discussion on the Correlation Between Family Socioeconomic Status and Parental Rearing Styles

From the perspective of the correlation between the two, family socioeconomic status is mainly related to the parents’ emotional warmth and understanding. The family socioeconomic status of Jingzhou primary school students is significantly related to their parents’ emotional warmth and understanding, while the family socioeconomic status of Shanghai primary school students is only significantly related to their father’s emotional warmth and understanding. This difference also directly led to the subsequent differences in the mediating effect of parental rearing styles between the two regions.

There is another point worth noting here, that is, there is no significant correlation between family socioeconomic status and other dimensions of parental rearing styles. This may be due to the fact that this study only conducted a survey in one primary school in both regions. Because the students in the same primary school live in the same village or community, even if the family economic level is different, they are likely to be very similar in social culture. So there are many similarities in the way of parenting.

#### The Correlation Between Family Socioeconomic Status and Emotional Intelligence

The family socioeconomic status and emotional intelligence of primary school students in the two regions are both significantly positively correlated, which is consistent with the fact that Zhu and Zhang (2013) found that low socioeconomic status has a negative impact on children’s social and emotional development. And the interesting point is that the correlation coefficients of the two regions are very close (0.23 and 0.24). This finding can still be explained by the fact that the families of primary school students live in the same village or the same community. Although the socioeconomic status of families in the same village or community has differences, but not too large, so the effect size may also be similar, which is also in line with Hansen's (2003) study that the degree of social stratification determines the size of the SES effect.

#### The Correlation Between Parenting Style and Emotional Intelligence

##### The Basic Role of Emotional Support for Children’s Development

The emotional intelligence of Jingzhou primary school students is only significantly related to the dimension of parents’ emotional warmth and understanding. In contrast, the emotional intelligence of Shanghai primary school students is related to more dimensions of parenting styles. This is actually in line with the theory of developmental psychology and clinical psychology for children’s emotional development. Bowlby’s “attachment” theory (Bowlby, 1969) and Winnicott’s “holding” theory (Winnicott, 1992) both emphasize that emotional support from infants to childhood is crucial to the development of children’s emotions. Only on the basis of sufficient emotional support, children can smoothly develop and improve their personality structure and behavior. Since the emotional warmth and understanding of Jingzhou primary school students’ parents are significantly lower than that of Shanghai primary school students, the need for this most important emotional support and parenting style may mainly determine the development of their emotional intelligence. While for Shanghai primary school students, the influence of other parenting styles on emotional intelligence may be gradually revealed based on the relatively sufficient parents’ emotional warmth and understanding.

##### The Positive Correlation Between Mother’s Excessive Interference and Overprotection With Emotional Intelligence

In addition, there is a correlation that may be inconsistent with previous studies, that is, emotional intelligence is positively correlated with mothers’ excessive Interference and overprotection. Excessive intervention and protection by parents is not conducive to the independence and development of children, which is the conclusion of many western studies (Arrindell et al., 1983). However, it should be noted that this view is based on the view that children are independent of their parents as the main development goal in the context of Western society and culture, interference, and protection is certainly not conducive to the improvement of its independence. However, in the context of Chinese society and culture, interference, and protection on the one hand hinder children’s independence, on the other hand it is also a manifestation of love. For example, “Zhan Guo Ce · Zhao Ce” said, “Parents’ beloved sons will have far-reaching plans.” At the same time, the traditional Chinese culture of “filial piety” requires children to consider and obey their parents’ ideas to a certain extent. For example, “Book of Rites · Nei Ze” said: “As a filial son, you should make your parents happy without going against their wishes.” It can be seen that the independence of children from their parents in traditional Chinese culture is not encouraged. And the symbol of children’s adulthood is mentioned in the opening chapter of “Book of Rites·Guanyi”: “The reason why a person is a person is also ritual and righteous. The beginning of ritual and righteousness lies in correcting the body, correcting the attitude, and making the speech decently. The body is correct, the color is correct, the speech is decent, and then, a person is ritual and righteous. Making the father and son a close relationship.” This view may indicate that a good relationship with parents, rather than complete independence, is the main goal of children’s growth and development. Appropriate speech is also strongly related to emotional intelligence, which may provide a basis for the positive correlation between this education method and primary school students’ emotional intelligence. In addition to the basis of traditional literature, a research on ordinary people and neurotic patients in modern China found that the main differences between their parenting styles are emotional warmth and understanding, punishment and harshness, and rejection and denial, while there is no significant difference in the three parenting styles: father’s excessive interference and father’s overprotection, mother’s excessive interference, and overprotection (Yue, 1993). It can be used as another evidence that this kind of education does not necessarily have only negative effects in China. It is true that in the process of China’s modernization, the growth and maturity of children, relative independence from their parents, has become an increasingly important sign, but the influence of traditional culture still exists and continues to be passed on. Huo (2000) also proposed a “complex of generation,” which different from Freud’s “Oedipus complex,” to reveal the dialectical contradictory structure of the dependence and independence of Chinese offspring on their parents. The negative and positive significance of this impact is not the focus of this study, but it still provides a new perspective, that is, whether the parenting styles, such as excessive interference and overprotection, can be directly incorporated into the negative parenting style in China like the West. Which is a question worthy of further discussion.

### Discussion on the Mediating Effect of Parenting Styles of Primary School Students in Different Regions in the Influence of Family Socioeconomic Status on Emotional Intelligence

In the results of the mediating effect above, it can be seen that the emotional warmth and understanding of the father and mother of Jingzhou primary school students play a part of the mediating effect in the influence of family socioeconomic status on emotional intelligence, while there is a completely mediating effect between the two variables by Shanghai primary school students’ fathers’ emotional warmth and understanding. This difference is also consistent with the related differences in the socioeconomic status of families and parental rearing styles in the above two regions, and we will discuss them here.

#### Discussion on the Difference of Parental Emotional Support on Children’s Emotional Development

This issue involves parents’ emotional warmth and understanding of parenting style. Early child psychologists mostly emphasized the importance of mother’s emotional support based on actual experience. For example, Melanie Klein put forward “Good Mother, bad mother” theory; Winnicott emphasizes “good enough mother”; Bowlby’s earliest attachment theory also starts with the mother–child relationship. However, with the further development of research, the role of father’s emotional support has gradually been emphasized and explored. The multiple attachment theory holds that the attachment of infants to their father is almost as much as that of their mothers in the second half of their first year of age. The emotional support of father and mother may each play an irreplaceable role in the development of infants and young children. The mother is the baby’s main caregiver, and the father is the child’s playmate (Wang, 2018).

Therefore, the emotional warmth and understanding parenting style of the parents of Jingzhou primary school students plays a part of the intermediary role. On the one hand, family socioeconomic status has a significant impact on this parenting style, on the other hand, the increase in emotional support of either side of the father and the mother may have an important impact on the child’s emotional intelligence. For Shanghai primary school students, family socioeconomic status only has a significant impact on father’s emotional warmth and understanding, but not on mothers. It may be that Shanghai mothers prefer to use emotional warmth and understand parenting methods, so family socioeconomic status has no significant impact on it. The father’s emotional warmth and understanding play a complete mediating effect. It may be that as the family’s socioeconomic status improves, fathers pay more attention to the care and emotional support of their children (Li et al., 2006). When the mother’s emotional warmth and understanding are adequate, the father’s emotional warmth and understanding may play a unique and critical role.

While we test the total sample, we find the father’s emotional warmth and understanding of total primary school students plays a completely mediating effect in the influence of family socioeconomic status on emotional intelligence, and the mother’s emotional warmth and understanding of total primary school students plays a part of the mediating effect in the influence of family socioeconomic status on emotional intelligence. It also shows that the father’s and mother’s emotional warmth and understanding play different roles in the development of children’s emotional intelligence.

### Research Limitations and Follow-Up Research Recommendations

#### Limitations

##### Research Subjects

This study only investigated two schools in Shanghai and Jingzhou. Although they are representative, there are still problems, such as insufficient sample size and limited sampling range. And it leads to a certain similarity in family socioeconomic status, and the stratification effect is not obvious enough.

##### Research Factors

This research focuses on the relationship between family socioeconomic status, parenting styles, and emotional intelligence. However, there are still some factors that have not been taken into consideration, such as the differences in school education levels mentioned in the article.

##### Research Method

This research mainly adopts the method of horizontal comparative research, without the support of longitudinal research, it is impossible to explore the longitudinal influence mechanism and time effect between several variables.

#### Recommendations

1. The follow-up research can be aimed at the deficiencies of this article and carry out targeted supplementary research, such as expanding the sample size and sample range, adding variables, such as school education level for research, and increasing longitudinal research.
2. Follow-up research can be implemented on the basis of theoretical research to implement practical interventions, such as parent education to improve parenting styles, or direct psychological interventions to primary school students to promote the healthy development of children’s emotional intelligence.
3. Follow-up research can study some important issues that have not been fully discussed in the article, such as the unique mechanism of the children’s growth and development in the family under the Chinese cultural background.

## Conclusion

1. The family socioeconomic status, parenting style, and emotional intelligence of primary school students all differ significantly by region. The family socioeconomic status and emotional intelligence of primary school students in Jingzhou are significantly lower than those of primary school students in Shanghai. In terms of parenting styles, the father’s emotional warmth and understanding and the mother’s emotional warmth and understanding are also significantly lower in Jingzhou than in Shanghai; in addition, the father’s excessive interference is significantly lower in Jingzhou than in Shanghai; the other three dimensions of father’s parenting styles, punishment and harshness, overprotection, and rejection and denial, are all significantly higher in Jingzhou than in Shanghai. The other three dimensions of mother’s parenting style, punishment and harshness, excessive interference and overprotection, and rejection and denial, are not significantly different from each other.
2. Primary school students family socioeconomic status, parenting style, and emotional intelligence are significantly correlated. For primary school students in Jingzhou, their family socioeconomic status is significantly and positively related to emotional intelligence, father’s emotional warmth and understanding, and mother’s emotional warmth and understanding; and their emotional intelligence is also significantly and positively related to father’s emotional warmth and understanding and mother’s emotional warmth and understanding. In contrast, for Shanghai primary school students, family socioeconomic status is significantly and positively related to emotional intelligence and father’s emotional warmth and understanding only; while emotional intelligence is significantly positively related to father’s emotional warmth and understanding, mother’s emotional warmth and understanding, and mother’s excessive interference and overprotection on the one hand, and is significantly negatively related to father’s punishment and harshness, father’s rejection and denial, and mother’s punishment and harshness on the other hand.
3. Primary school student’s parenting style plays mediating effect between family socioeconomic status and emotional intelligence, but there are regional differences in this effect. The Jingzhou primary school students’ father’s and mother’s emotional warmth and understanding parenting style both play a part mediating effect between family socioeconomic status and emotional intelligence, while the Shanghai primary school students’ father’s emotional warmth and understanding parenting style plays a fully mediating effect between family socioeconomic status and emotional intelligence.

## Data Availability Statement

The original contributions presented in the study are included in the article/Supplementary Material, further inquiries can be directed to the corresponding author.

## Ethics Statement

The studies involving human participants were reviewed and approved by Ethical Board of the Psychological Faculty of the Fudan University. The patients/participants provided their written informed consent to participate in this study. Written informed consent to participate in this study was provided by the participants’ legal guardian/next of kin.

## Author Contributions

ZL and GW contributed to conception and design of the research and conducted the research. ZL performed the statistical analysis, supervised by GW. ZL wrote the first draft of the manuscript. All authors contributed to manuscript revision, read, and approved the submitted version.

## Conflict of Interest

The authors declare that the research was conducted in the absence of any commercial or financial relationships that could be construed as a potential conflict of interest.

## Publisher’s Note

All claims expressed in this article are solely those of the authors and do not necessarily represent those of their affiliated organizations, or those of the publisher, the editors and the reviewers. Any product that may be evaluated in this article, or claim that may be made by its manufacturer, is not guaranteed or endorsed by the publisher.
